# Transcriptomic analyses suggest a dominant role of insulin in the coordinated control of energy metabolism and ureagenesis in goat liver

**DOI:** 10.1186/s12864-019-6233-9

**Published:** 2019-11-14

**Authors:** Zhongyan Lu, Zhihui Xu, Zanming Shen, Hong Shen, Jörg R. Aschenbach

**Affiliations:** 1 Key Lab of Animal Physiology and Biochemistry, College of Veterinary Medicine, Nanjing Agricultural University, Nanjing, Jiangsu China; 2 College of Life Science, Nanjing Agricultural University, Nanjing, Jiangsu China; 3 Bioinformatics Center, Nanjing Agricultural University, Weigang No.1, Nanjing, 210095 Jiangsu China; 4 Institute of Veterinary Physiology, Freie Universität Berlin, Berlin, Germany

**Keywords:** Ureagenesis, Liver, Fatty acid anabolism, Insulin signaling, Endocrine regulation

## Abstract

**Background:**

The ureagenesis plays a central role in the homeostatic control of nitrogen metabolism. This process occurs in the liver, the key metabolic organ in the maintenance of energy homeostasis in the body. To date, the understanding of the influencing factors and regulators of ureagenesis in ruminants is still poor. The aim of this study was to investigate the relationship between energy metabolism and ureagenesis and detect the direct regulators of ureagenesis in the liver by using RNA-seq technology.

**Results:**

Eighteen four-month-old male goats were divided into two groups randomly and received a diet containing 10% (LNFC group, *n* = 9) or 30% non-fiber carbohydrate (MNFC group, *n* = 9), respectively, for four weeks. The global gene expression analysis of liver samples showed that, compared with a LNFC diet, the MNFC diet promoted the expression of genes required for synthesis of fatty acid and glycerol, whereas it suppressed those related to fatty acid oxidation, gluconeogenesis from amino acids and ureagenesis. Additionally, gene expression for rate-limiting enzymes of ureagenesis were highly correlated to the gene expression of key enzymes of both fatty acid synthesis and glycerol synthesis (Spearman correlation coefficient > 0.8 and *p* < 0.05). In the differentially expressed signaling pathways related to the endocrine system, the MNFC diet activated the insulin and PPAR signaling pathway, whereas it suppressed the leptin-JAK/STAT signaling pathway, compared with the LNFC diet. Reverse transcription quantitative PCR analyses of 40 differentially expressed genes confirmed the RNA-seq results (R^2^ = 0.78).

**Conclusion:**

Our study indicated that a dietary NFC-induced increase of energy supply promoted lipid anabolism and decreased ureagenesis in the caprine liver. By combining our results with previously published reports, insulin signaling can be suggested to play the dominant role in the coordinated control of hepatic energy metabolism and ureagenesis.

## Background

Ureagenesis is the major way of eliminating the nitrogenous waste of protein metabolism in mammals and, especially, in ruminants. It prevents ammonia intoxication and contributes to hepatic pH homeostasis by converting ammonia (derived from gastrointestinal absorption and amino acid (AA) systemic turnover) and bicarbonate into urea. Concomitantly, 40–80% of liver-synthesized urea (LSU) is recycled back to the rumen, providing a valuable source of N for the bacterial synthesis of host-utilizable protein as a urea N salvage mechanism [1]. Accordingly, ureagenesis plays a significant role in the homeostatic control of N metabolism and in the sustenance of life and health in ruminants. On this basis, considerable interest has been generated with regard to the understanding of the influencing factors and regulators of ureagenesis in ruminants in order to refine strategies for the promotion of animal health and the enhancement of animal productivity.

To date, the factors that influence the ureagenesis of ruminants remain poorly understood. Hepatic infusion studies have shown that an increase of ammonia or AA content promotes LSU in sheep [2]. Other hepatic infusion and hepatocyte incubation studies have demonstrated that the concentration of propionate affects LSU, although in vivo and in vitro studies have revealed opposite results [3–5]. In addition, our understanding of the regulators of ureagenesis is primarily based on studies that have been carried out on monogastric animals, suggesting that hormones, such as glucagon, glucocorticoids and insulin, are the direct regulators of ureagenesis [6–8]. Whether endocrine regulation in ruminants is similar to that of monogastric animals has not been studied.

Ureagenesis occurs in the liver, which is the key metabolic organ maintaining energy homeostasis in the body. We speculate that hepatic ureagenesis is affected by hepatic energy metabolism for the following reasons. (1) A tight crosstalk exists in the ureagenesis and energy metabolism of the liver. For example, acetyl-CoA, the precursor for N-acetyl-glutamate (NAG), which affects ureagenesis by activating carbamoyl phosphate synthase-1 (EC: 6.3.4.16; CPS1), serves as the precursor for the de novo synthesis of fatty acid (FA). Both oxaloacetate and α-ketoglutarate (α-KG), which assist the delivery of ammonia during ureagenesis, can enter the tricarboxylic acid (TCA) cycle. (2) Ureagenesis is an energy-requiring reaction. Four to eight molecules of adenosine triphosphate (ATP) are required to generate one molecule of urea depending on N source [9]. (3) Glutamine, the major energy substrate for small intestinal tissue in ruminants [10], is the second way by which the liver eliminates excess ammonia, with the assistance of renal glutaminase. A study in the rat liver has shown that glutamine production is inversely related to urea production [11].

Dietary non-fiber carbohydrate (NFC), a rapid fermentable substrate for the microbiota of the rumen, might promote the generation and epithelial absorption of ruminal short-chain fatty acids (SCFAs), which account for 50–75% of the total metabolizable energy of ruminant feed-stuff [12]. Studies in the Holstein heifer have demonstrated that changes of dietary NFC affected the energy metabolism in the liver [13]. In order to study the relationship of hepatic energy metabolism and ureagenesis in vivo and to concomitantly investigate the potential regulators of the ureagenesis in ruminants, we have collected liver samples from goats receiving a 10% or 30% NFC diet. By applying transcriptome sequencing and bioinformatics analysis, we have constructed the pathways related to hepatic energy metabolism and hepatic ureagenesis, tested the differential expression of these pathways among groups, and additionally, analyzed correlations between gene expression for enzymes of energy metabolism and that of rate-limiting enzymes of ureagenesis, i.e., CPS1, glutamic-oxaloacetic transaminase 1 (EC: 2.6.1.1; GOT1), and argininosuccinate synthase 1 (EC: 6.3.4.5; ASS1) [14]. Furthermore, we aimed at identifying hormone candidates which potentially regulate the ureagenesis in goats fed with different ratios of NFC.

## Results

### Differences of ruminal SCFAs concentrations among groups

Compared with the goats fed with a diet containing 10% NFC (LNFC group), the concentrations of ruminal butyrate, propionate, and total SCFAs were significantly increased, whereas ruminal pH was significantly decreased in the goats fed with a diet containing 30% NFC (MNFC group). Ruminal acetate did not show any significant differences among groups (Table 1).

**Table 1:** Dietary effect on ruminal SCFA concentrations and ruminal pH

Item	LNFC^1^	MNFC^1^	*P*^2^
pH	6.9 ± 0.07	6.5 ± 0.12	0.03
Acetate, mM	42.7 ± 2.31	46.2 ± 2.24	0.09
Propionate, mM	13.3 ± 0.40	23.1 ± 0.93	0.01
Butyrate, mM	7.1 ± 0.81	9.4 ± 0.69	0.03
Total SCFA^3^, mM	63.1 ± 4.10	78.7 ± 6.84	0.01

^1^Values are means ± standard error

^2^ *p* value in the two-tailed T-test

^3^ Total SCFA = Acetate + Propionate + Butyrate

### Differences of hepatic gene expression profiles among groups

After filtering, 38.56 ± 1.19 million of clean reads were obtained per sample (Additional file 1). In the sequencing alignment, 90.44 ± 0.37% of the clean reads per sample were mapped to the caprine reference genome. A total of 12,365 genes with fragments per kilobase of exon model per million reads mapped (FPKM) > = 1 in at least one sample were identified in the RNA-seq data. Among them, 1153 genes were identified to be differentially expressed genes (DEGs) between the groups (Additional file 2). Systematic cluster analysis showed that the 18 samples were categorized into two clusters according to the ratio of dietary NFC received by the goats (Additional file 3).

### Differences of ureagenesis at the transcriptional level among groups

Ureagenesis is known to involve three mitochondrial enzymes, i.e., CPS1, GOT1, and ornithine transcarbamylase (EC 2.1.3.3; OTC), three cytosolic enzymes, i.e., ASS1, argininosuccinate lyase (EC 4.3.2.1; ASL), and arginase (EC 3.5.3.1; ARG), and one mitochondrial cofactor-producing enzyme, i.e., NAG synthase (EC 2.3.1.1; NAGS). Within liver mitochondria, two ways have been suggested that supply NH_3_ and NH_2_-N to ureagenesis. The first involves glutaminase (EC 3.5.1.2; GLS), which releases ammonia and glutamate from glutamine. The second involves glutamate dehydrogenase (EC 1.4.1.3; GDH), which converts glutamate into ammonia and α-KG, or vice versa [14]. In addition, certain AA might be converted into glutamate by transaminases, providing NH_2_-N to ureagenesis. Based on this knowledge and the gene expression profiles revealed in this study, we constructed the pathway of ureagenesis, and furthermore, determined the significantly expressed genes along this metabolic pathway.

**Fig. 1:**
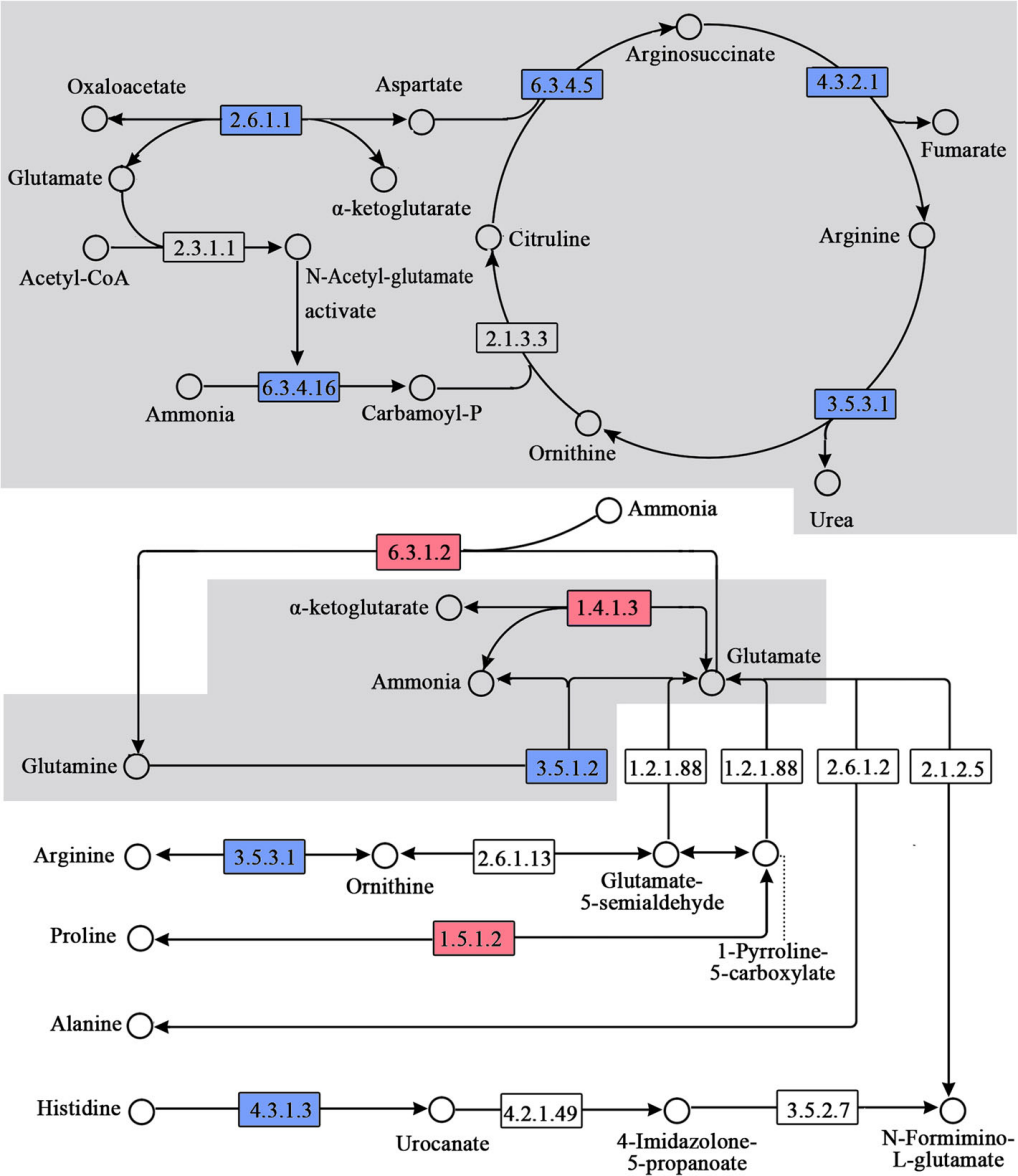
Ureagenesis-related pathways reconstructed in present study. The numbers in boxes represent the EC numbers of pathway enzymes. The box in red refers to the enzymes whose expression was significantly upregulated in the MNFC group compared with the LNFC group. The box in blue refers to the enzymes whose expression was significantly downregulated in the MNFC group compared with the LNFC group. The gray background indicates the main route of ureagenesis. This figure was modified from the KEGG pathway maps chx00220 and chx250. The written permission was obtained from the Kanehisa Laboratories to use and adapt these KEGG pathway maps

As shown in Fig. 1, the gene expression of 5/7 enzymes along the main route of ureagenesis was significantly decreased in the MNFC group in comparison with the LNFC group (Table 2). Moreover, the expression of ureagenesis pathway (main route) was significantly different among groups according to the kyoto encyclopedia of genes and genomes (KEGG) enrichment analysis (Additional file 4). With regard to the supply of ammonia / NH_2_-N to ureagenesis, the expression of GLS was significantly decreased, whereas the expression of GDH was significantly increased in the MNFC group compared with the LNFC group. On the branching routes of ureagenesis, the expression of ARG and that of histidine ammonia lyase (EC 4.3.1.3; HAL) were significantly decreased, whereas the expression of glutamate-ammonia ligase (EC 6.3.1.2; GLUL) and that of pyrroline-5-carboxylate reductase 1 (EC 1.5.1.2; PYCR1) were significantly increased in the MNFC group compared with the LNFC group (Table 2). Details of the pathway enzymes shown in Fig. 1 are listed in Additional file 5.

**Table 2:** Differentially expressed genes on the pathways of energy metabolism and ureagenesis in the goat liver

Item	Gene Description	EC	LNFC^a^	MNFC^a^
Energy metabolism	fatty acid synthase	2.3.1.85	1.65 ± 0.11	14.87 ± 0.17
	HMGCS1	2.3.3.10	15.02 ± 0.28	101.41 ± 6.06
	ACSS2	6.2.1.1	6.92 ± 0.43	31.98 ± 0.72
	acetyl-CoA carboxylase alpha	6.4.1.2	1.77 ± 0.09	6.50 ± 0.12
	ATP citrate lyase	2.3.3.8	2.88 ± 0.22	7.19 ± 0.37
	acetyl-CoA carboxylase beta	6.4.1.2	3.59 ± 0.25	8.29 ± 0.22
	enolase family member 4	4.2.1.11	1.35 ± 0.18	2.94 ± 0.12
	AKR1B1	1.1.1.21	2.46 ± 0.17	5.21 ± 0.82
	ALDH7A1	1.2.1.3	42.70 ± 0.63	89.68 ± 2.81
	citrate synthase	2.3.3.1	4.01 ± 0.09	8.07 ± 0.15
	acyl-CoA dehydrogenase	1.3.8.9	353.15 ± 15.80	176.21 ± 4.05
	acetyl-CoA acetyltransferase 1	2.3.1.9	377.39 ± 13.05	187.99 ± 7.42
	acyl-CoA oxidase 1	1.3.3.6	94.65 ± 2.52	46.41 ± 0.49
	acyl-CoA synthetase	6.2.1.3	67.04 ± 1.77	32.23 ± 0.64
	enoyl-CoA hydratase	1.1.1.211	83.85 ± 0.67	39.79 ± 0.47
	malonyl-CoA decarboxylase	4.1.1.9	61.83 ± 3.32	28.86 ± 1.55
	fructose-bisphosphatase 2	3.1.3.11	3.63 ± 0.17	1.69 ± 0.32
	carnitine palmitoyltransferase 2	CPT2	39.61 ± 2.37	17.92 ± 0.56
	aldolase, fructose-bisphosphate B	4.1.2.13	4266.95 ± 240.93	1924.19 ± 82.61
	carnitine palmitoyltransferase 1B	CPT1	2.91 ± 0.24	1.29 ± 0.09
	ACSM1	6.2.1.2	801.14 ± 21.60	343.51 ± 10.80
	acetyl-CoA acyltransferase 1	2.3.1.16	1292.50 ± 70.23	467.31 ± 11.32
	carnitine palmitoyltransferase 1A	CPT1	44.81 ± 2.06	14.18 ± 0.31
	lactate dehydrogenase B	1.1.1.27	343.71 ± 35.60	107.23 ± 8.80
	acyl-CoA thioesterase 12	3.1.2.1	3.68 ± 0.31	1.13 ± 0.08
	glucose-6-phosphatase	3.1.3.9	187.53 ± 1.98	55.81 ± 0.83
	pyruvate carboxylase	6.4.1.1	312.03 ± 11.05	91.19 ± 1.30
	fructose-bisphosphatase 1	3.1.3.11	1307.74 ± 57.02	312.13 ± 12.39
	HMGCS2	2.3.3.10	841.12 ± 26.52	131.76 ± 4.35
Ureagenesis	pyrroline-5-carboxylate reductase 1	1.5.1.2	5.27 ± 0.90	50.95 ± 1.83
	glutamate dehydrogenase 1	1.4.1.3	377.55 ± 10.60	766.01 ± 25.12
	glutamate-ammonia ligase	6.3.1.2	92.93 ± 0.93	185.03 ± 9.79
	argininosuccinate lyase	4.3.2.1	654.00 ± 24.75	326.93 ± 9.07
	carbamoyl-phosphate synthase 1	6.3.4.16	519.96 ± 3.93	257.68 ± 5.98
	argininosuccinate synthase 1	6.3.4.5	2293.04 ± 88.68	1134.59 ± 28.93
	glutamic-oxaloacetic transaminase 1	2.6.1.1	89.37 ± 3.70	44.02 ± 0.50
	arginase 2	3.5.3.1	1.71 ± 0.32	0.77 ± 0.28
	arginase 1	3.5.3.1	853.86 ± 49.01	375.18 ± 32.10
	glutaminase 2	3.5.1.2	79.93 ± 2.13	35.07 ± 1.18
	histidine ammonia-lyase	4.3.1.3	47.15 ± 0.38	18.18 ± 1.16

HMGCS1:3-hydroxy-3-methylglutaryl-CoA synthase 1; ACSS2: acyl-CoA synthetase short-chain family member 2; AKR1B1: aldo-keto reductase family 1 member B; ALDH7A1: aldehyde dehydrogenase 7 family member A1; ACSM1: acyl-CoA synthetase medium-chain family member 1; HMGCS2: 3-hydroxy-3-methylglutaryl-CoA synthase 2. ^a^ values are mean value ± standard error

### Differences of hepatic energy metabolism mode among groups

Ruminants absorb little glucose from the intestine and many energy-consuming processes rely on short and long-chain FAs, triacylglycerols (TAG) and ketone bodies. Thereby, endogenous glucose that is generated primarily through hepatic gluconeogenesis can be partially spared for tissues that have a specific glucose demand, such as the brain cells in the central nervous system. Propionate complemented by lactate, glycerol, and AAs is the major precursor for hepatic gluconeogenesis [15]. Based on this knowledge and the gene expression profiles obtained in this study, we determined the energy metabolism profile and pin-pointed the differentially expressed genes among the groups.

**Fig. 2:**
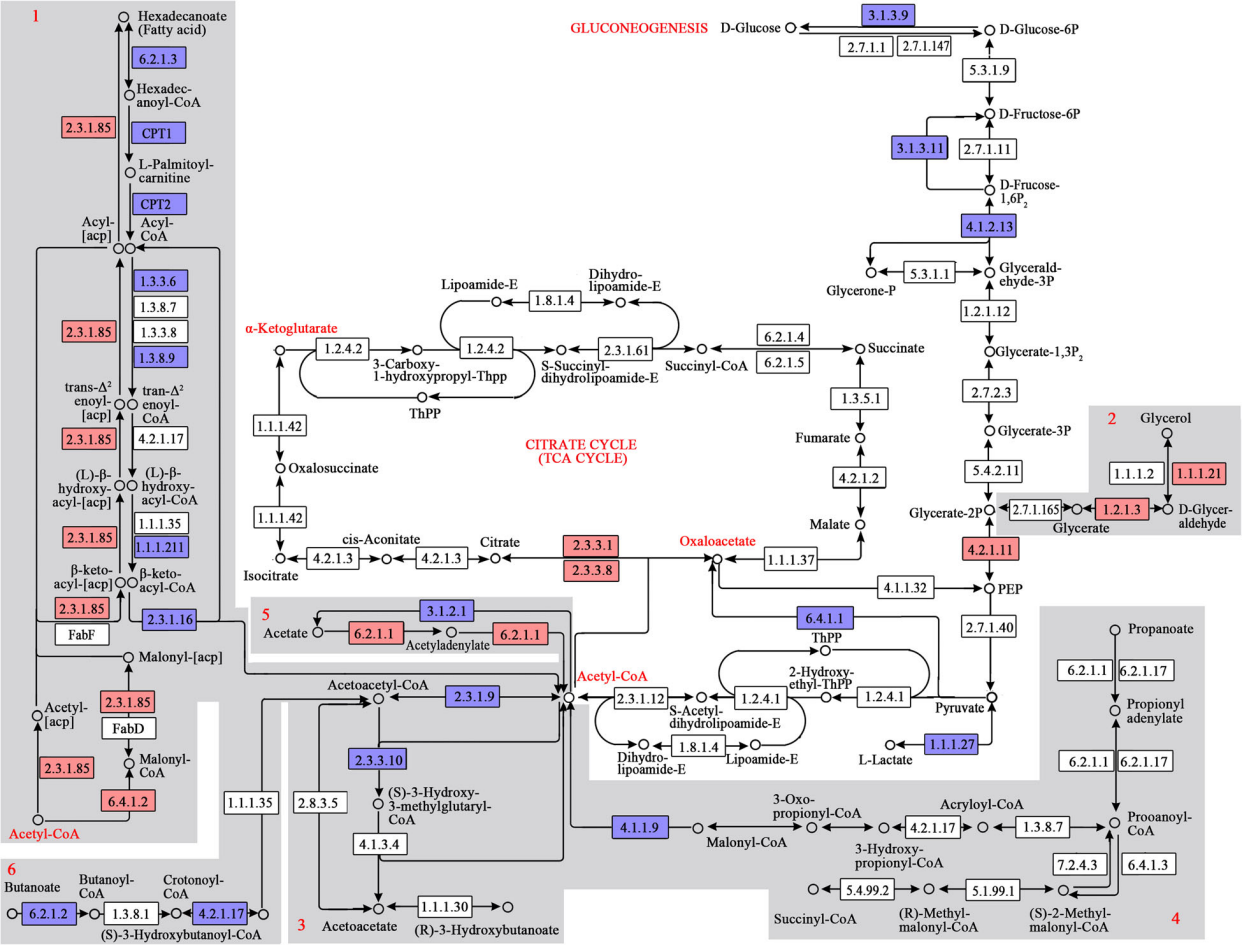
Pathways of hepatic energy metabolism reconstructed in the present study. The numbers in boxes represent the EC numbers of the pathway enzymes. The box in red refers to the enzymes whose expression was significantly upregulated in the MNFC group compared with the LNFC group. The box in blue refers to the enzymes whose expression was significantly downregulated in the MNFC group compared with the LNFC group. 1 refers to the pathway of glycerol synthesis; 2 refers to fatty acid metabolism; 3 refers to ketone body metabolism; 4 refers to propanoate metabolism; 5 refers to acetate metabolism; and 6 refers to butanoate metabolism. The shared substrates of ureagenesis and energy metabolism are marked in red. This figure was modified from the KEGG pathway maps chx00010, chx00020, chx00030, chx00061, chx00071, chx00072, chx00561, chx00620 and chx00640. The written permission was obtained from the Kanehisa Laboratories to use and adapt these KEGG pathway maps

As shown in Fig. 2, the gene expression of the pathway enzymes of FA biosynthesis, of the enzymes responsible for the conversion between glycerol and phosphoenolpyruvate (PEP), of the citrate-shuffle enzymes, i.e., citrate synthase in mitochondria (EC 2.3.3.1, CS) and citrate lyase in the cytoplasm (EC 2.3.3.8, CL), and of the enzymes responsible for the conversion from acetate to acetyl-CoA was significantly upregulated, whereas the gene expression of the pathway enzymes of FA degradation, of enzymes responsible for the conversion of acetyl-CoA to acetate, and of enzymes responsible for the conversion of butanoate to acetoacetyl-CoA was significantly downregulated in the MNFC group compared with the LNFC group (Table 2). In addition, the gene expression of three key enzymes in the gluconeogenesis, i.e., pyruvate carboxylase (EC 6.4.1.1; PC), fructose-bisphosphatase (EC 3.1.3.11; FBP), and glucose-6-phosphatase (EC 3.1.3.9; G6PC3), together with aldolase (EC 4.1.2.13), was significantly downregulated. The gene expression of the cytosolic form of 3-hydroxy-3-methylglutaryl-CoA synthase 1 (EC 2.3.3.10; HMGCS1), which is a key enzyme in cholesterol biosynthesis, was significantly upregulated; whereas, the mitochondrial HMGCS2, which has a rate-limiting role in ketone body production, was significantly downregulated in the MNFC group compared with the LNFC group (Table 2). The details of the pathway enzymes shown in Fig. 2 are listed in Additional file 5.

### Highly related enzymes of energy metabolism pathways and ureagenesis

As shown in Additional file 6, four enzymes of FA degradation and three key enzymes of gluconeogenesis were positively related to at least one of the rate-limiting enzymes of ureagenesis, whereas FA synthase (EC: 2.3.1.85; FASN) of FA biosynthesis was negatively related to the rate-limiting enzymes of ureagenesis at the transcriptional level. In addition, FASN was negatively related to the three enzymes of FA degradation and to three key enzymes of gluconeogenesis at the transcriptional level. Moreover, the key enzymes of gluconeogenesis were positively related to six enzymes of FA degradation and negatively related to the enzymes of the glycerol synthesis pathway and citrate-shuffle enzymes at the transcriptional level. Finally, the enzymes of glycerol synthesis were positively related to the citrate shuffle enzymes at the transcriptional level.

### Differentially expressed signaling pathways

To identify hormonal signals that could explain the alterations in metabolism, we checked the results of the KEGG enrichment analysis, which showed that, within the endocrine system, the activities of peroxisome proliferators activated receptor (PPAR) signaling pathway, insulin signaling pathway and adipocytokine signaling pathway were significantly changed among the groups (Additional file 4). On checking the expression levels of differentially expressed genes on these pathways individually, we found that most of them were upregulated (*p* < 0.05), and only the leptin receptor (LEP), janus kinase 3 (JAK3), and signal transducer and activator of transcription 3 (STAT3) in the adipocytokine signaling pathway were significantly downregulated in the MNFC group compared with the LNFC group.

### Confirmation of RNA-seq results by RT-QPCR

**Fig. 3:**
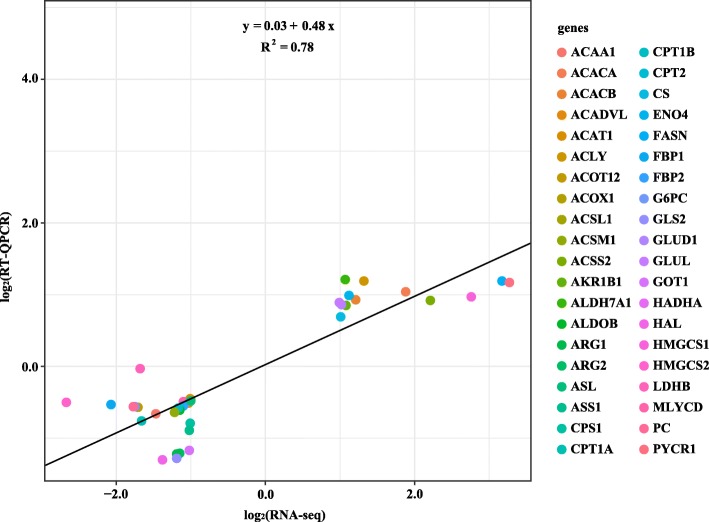
Linear regression analysis of relationships between RNA-seq results and RT-QPCR results for 40 selected genes

To validate RNA-seq results, we compared the expression of 40 genes listed in Table 2 by using the RT-QPCR method, and the results are shown in Additional file 7. In general, the expression analyses of 39 genes verified the significant differences discovered by RNA-seq, whereas the expression of lactate dehydrogenase B (EC: 1.1.1.27; LDHB) showed no significant difference between the groups in the RT-QPCR analysis. Linear regression analysis showed that fold changes of RT-QPCR results were highly consistent with that of RNA-seq results (R^2^ = 0.78) (Fig. 3).

## Discussion

### Dietary NFC suppressed ureagenesis and promoted energy substrate stores in goat liver

In the present study, the MNFC diet suppressed ureagenesis, while promoting the conversion of ammonia into glutamate and the conversion of glutamate into glutamine in the goat liver, compared with the LNFC diet. This is consistent with the in vitro study of Haeussinger & Sies who have shown that glutamine production is negatively related to urea production in the rat liver [11]. In addition, the MNFC diet promoted hepatic FA synthesis and glycerol synthesis at 6 h after the morning feeding (the fed state), compared with the LNFC diet. This is consistent with a study in Holstein heifers in which a 40% concentrate diet upregulated lipid biosynthesis in the liver, in the fed state, compared with a 20% concentrate diet [13]. Alternative studies have shown that FA degradation is upregulated in the fasted state [16] or when increasing the forage concentration of the ration in Holstein heifers [17]. This regulation of hepatic FA turnover bears similarities to monogastric animals, where TAGs generated by the hepatocytes in the fed state are released into the circulation and metabolized by extrahepatic tissues in the fasted state [18]. However, different from the monogastric animals, SCFAs and ketone bodies from the rumen provide prominent substrates for FA synthesis and esterification in the adipose tissue of ruminants, which also contribute to the energy supply of extrahepatic tissues upon requirement [19]. Due to lower availability and several glucose-sparing mechanisms, glucose is used less for the energy supply of ruminants compared to monogastric species [15]. However, glutamine is the major energy substrate for small intestinal tissues in both ruminants and non-ruminants. Together, our results indicate that dietary NFC favors nitrogen conservation in AAs and suppresses hepatic ureagenesis while simultaneously promoting the synthesis of lipid energy stores in the fed state in ruminants.

### Hepatic nitrogen conservation implies coordinated changes in gluconeogenesis and ureagenesis

In the present study, gluconeogenesis and the conversion of lactate to oxaloacetate were downregulated, whereas the pathway for the conversion of propionate into oxaloacetate exhibited no significant change in the MNFC group compared with the LNFC group. Among the precursors of hepatic gluconeogenesis, propionate absorbed from the gastrointestinal tract plays the most important role in hepatic gluconeogenesis in ruminants. Larger amounts of AAs (both endogenous and exogenous AAs) support gluconeogenesis primarily when the supply of propionate and other precursors (such as lactate and pyruvate) is inadequate [15]. The latter partially applies when ruminants are fed a high-roughage diet [17].

In the present study, the ruminal production of propionate was increased, and simultaneously, the hepatic pathway for its conversion into oxaloacetate showed no significant change in the MNFC group. On the other hand, PC as a key entry point for lactate and small neutral AAs into gluconeogenesis was downregulated together with the two rate-limiting ‘exit enzymes’ of gluconeogenesis, FBP and G6PC3. This can be interpreted as a gradual switch of gluconeogenesis from a pull mechanism for propionate, lactate and AAs to a push mechanism driven primarily by the increased availability of propionate. A previous study in dairy cows similarly evidenced a decreased mRNA abundance and enzyme activity of PC in dairy cows when surplus supply of energy was achieved by continuous intravenous glucose infusions. In that study, an increase of energy supply by 30% led to a drop of plasma urea concentration by 20% [20]. Accordingly, we infer that both an increase in propionate supply due to increased NFC feeding and a decreased requirement for glucose production due to intravenous glucose infusion lead to decreased AAs utilization for gluconeogenesis, and consequently, a decreased conversion of AA-NH_2_ into urea. This postulate is further supported by the decreased expression of pathways converting arginine and histidine into glutamate and by the results of our correlation analysis. The latter showed a positive correlation of the key enzymes of gluconeogenesis with the rate-limiting enzymes of ureagenesis (Spearman correlation coefficient (SCC) > 0.8 and *p* < 0.05; Additional file 6).

### Increased TAG anabolism deductible from transcripts for glycerol synthesis, FA synthesis and FA oxidation is linked to decreased ureagenesis

The generation of glycerol for TAG synthesis and gluconeogenesis are known to compete for the same precursors (i.e., AAs, propionate, and lactate), whose amounts are limited in the liver. Accordingly, it can be anticipated that an inverse relationship exists between glycerol synthesis and gluconeogenesis in the goat liver. This proposal is supported by the results of our correlation analysis, which demonstrated negative correlations of the key enzymes of glycerol synthesis with the key enzymes of gluconeogenesis (SCC > 0.8 and *p* < 0.05, Additional file 6).

Furthermore, our data showed that the synthesis pathway for FA was enhanced and their degradation pathway into acetyl-CoA was impaired in the MNFC group compared with the LNFC group. Ketone bodies and butyrate from the rumen and FA are known to be the major precursor for the generation of acetyl-CoA in the liver of ruminants, since acetate produced in rumen is poorly used by hepatocytes [21]. Accordingly, a decrease of FA degradation may decrease the generation of acetyl-CoA in the goat liver. Vice versa, our data also showed that the transportation of acetyl-CoA from the mitochondrion to the cytoplasm for FA synthesis, and the conversion pathway of acetyl-CoA into FA were enhanced in the MNFC group compared with the LNFC group. FA synthesis requires the supply of ATPs and reduced nicotinamide adenine dinucleotide phosphate (NADPH) by the TCA cycle, and acetyl-CoA is the precursor for the TCA cycle. Accordingly, the increase of FA synthesis requires also an increase the amount of acetyl-CoA entering the TCA cycle. This is supported by the increased expression of CS in the MNFC group, which is a pace-making enzyme of the TCA cycle [22].

The increase of FA synthesis may further suppress ureagenesis. The former may decrease the pool of acetyl-CoA in both mitochondrion and cytoplasm. Acetyl-CoA, in turn, is known to be the precursor for the synthesis of NAG, which is the activator of CPS1, the rate-limiting enzyme of ureagenesis. Therefore, if the acetyl-CoA utilized by FA synthesis is not adequately replenished from butyrate or ketone breakdown, a decreased pool of acetyl-CoA could lead to the decreased expression of CPS1, contributing to a decrease of ureagenesis in the MNFC group compared with the LNFC group. This is supported by the results of the correlation analysis showing high correlations of the key enzymes of FA synthesis and FA oxidation with the rate-limiting enzymes of ureagenesis (Additional file 6). Together, our data indicate that the increase of lipid energy reserves, such as the FA and TAG, can suppress liver ureagenesis by affecting NAG synthesis in goat liver.

### Insulin as prime signal to balance hepatic energy metabolism and ureagenesis

As a key metabolic organ in the body, the metabolic activities of liver are known to be tightly controlled by the neuroendocrine system. In the present study, a negative relationship was indicated between the hepatic synthesis of lipid energy substrates and ureagenesis. It appears highly likely that hormones directly regulate this relationship and thus maintain the balance between energy metabolism and ureagenesis in the goat liver. According to the KEGG enrichment analysis, within the endocrine system, the insulin and PPAR signaling pathway were activated, whereas the leptin-JAK/STAT signaling pathway (part of the adipocytokine pathway) was suppressed in the MNFC group compared with the LNFC group.

Insulin is a well-established hormonal regulator that promotes the FA synthesis and simultaneously suppresses FA oxidation [23, 24] and gluconeogenesis [15, 25] in the liver. A recent genome-wide association and transcriptomic study identified that polymorphism and transcription levels of CPT1 play a key role in body weight maintenance. The same study also identified three genes in the CPT1-glycine network that relate to diabetes and insulin signaling, thus supporting a central role for insulin in body weight maintenance via coordinated regulation of energy storage pathways and ureagenesis [26]. Therefore, the insulin signaling could plausibly explain the increase in hepatic FA synthesis and the simultaneous decreases in hepatic gluconeogenesis and ureagenesis. PPAR, specifically hepatic PPARα, signaling is known to suppress the expressions of genes encoding for AA metabolism and ureagenesis [27, 28]. However, this signaling is known to be induced by an increased concentration of hepatic FAs. Accordingly, PPARα signaling is supposed to be activated by the insulin signaling, further strengthening the regulatory effects of insulin on the FA metabolism and ureagenesis.

The leptin-JAK/STAT signaling pathway has previously been implicated to play an important role in the regulation of energy balance in mammals [29]. For example, leptin activates the JAK/STAT pathway in the hypothalamus to suppress the feed intake and promote lipolysis in the adipocytes of mice and pigs [30, 31]. Leptin-deficient (ob/ob) or LEP-deficient (db/db) mice showed an increased synthesis of FAs in the liver [32], and ob/ob mice had decreased expression of hepatic FA synthase upon leptin injection [33]. However, in contrast to the results of present study, the expressions of key genes of gluconeogenesis and ureagenesis were decreased in ob/ob mice upon leptin injection [33]. Therefore, the suppressed leptin-JAK/STAT signaling may explain the increase in FA synthesis but not the simultaneous decreases in gluconeogenesis and ureagenesis. A study in early lactation dairy cows showed that insulin suppressed the expression of hepatic LEP, leading to the decrease on ureagenesis [34]. Accordingly, we suggest that the leptin signaling pathway was suppressed by the insulin signaling to strengthen its promoting effects on the FA synthesis.

Taken together, we propose that the MNFC diet led to the activation of insulin signaling, which promoted the FA synthesis and simultaneously suppressed the ureagenesis in the goat liver. The increased storage of hepatic FAs then activated the PPAR signaling, which conversely enhanced the suppressing effects of insulin signaling on the ureagenesis. Besides, the insulin signaling induced the suppression of leptin signaling, which strengthened the promoting effects of insulin signaling on the FA synthesis.

## Conclusion

Liver is a key metabolic organ to govern energy metabolism. The gene expression profile of our study suggests that dietary NFC promotes the synthesis of FA and glycerol, while suppressing FA oxidation, gluconeogenesis from AAs and ureagenesis in the goat liver. Correlation analyses demonstrated that the rate-limiting enzymes of ureagenesis were highly correlated to key enzymes of FA and glycerol synthesis. These results suggest that the dietary NFC-induced increase of energy substrate stores is highly coordinated with the decrease of ureagenesis. The KEGG enrichment analyses suggested that insulin is likely the key signal for the coordinated alterations in FA metabolism and ureagenesis. The PPARα and leptin signaling triggered by the insulin signaling may strengthen the regulatory effects of insulin on these metabolic pathways.

## Methods

### Experimental design

Eighteen healthy goats (Boer × Yangtze River Delta White, male, 4 months, 14–16 kg bodyweight) used in the present study were obtained from the Liuhe Goat Farm (located in Nanjing, Jiangsu province, China), and then, maintained in the animal house of Nanjing Agricultural University. The goats were randomly allocated into two groups and received a 10% NFC diet (LNFC group, *n* = 9) or a 30% NFC diet (MNFC group, *n* = 9). Experimental diets were formulated according to the Feeding Standard of Meat-Producing Goats (NY/T816–2004). The ingredients and chemical compositions of the experimental diets are listed in Table 3.

**Table 3:** Dietary composition used in this study

Item	LNFC	MNFC
Ingredient, % of DM		
Guinea Grass	90	65
Corn meal	0	25
Soya bean meal	8	8
Mineral and vitamin supplement ^a^	1	1
Salt	0.25	0.25
CaHPO_4_	0.75	0.75
Chemical composition		
DM,%	91.4	89.9
Crude protein, %DM	10.3	10.7
Crude fat, %DM	3.5	3.4
Ash, %DM	4.7	3.9
NDF, %DM	66.8	50.7
NFC^b^, %DM	14.7	31.3

^a^Contained 16% calcium carbonate, 102 g/kg Zn, 47 g/kg Mn, 26 g/kg Cu, 1140 mg/kg I, 500 mg/kg Se, 340 mg/kg Co, 17,167,380 IU/kg vitamin A, 858,370 IU/kg vitamin D, and 23,605 IU/kg vitamin E

^b^ NFC = 100 – (NDF + CP + Crude fat + ash)

The goats were housed in individual tie-stall barns and had free access to water. To avoid the selection of dietary components and to maintain the desired ratio, a total mixed ratio (TMR) was offered at 0700 and 1800 daily for the 28-day experimental period, which followed a 14-day period dedicated to adaptation. Feed intake and all refusals of individual goats were measured daily during the experiment. The amount of diet offered during the experiment was adjusted on a weekly basis to allow about 10% orts. Feeds were sampled at the beginning and end of the experiment. The dry matter, ash, crude fat, and crude protein contents of samples were analyzed according to the procedures of AOAC [35]. The acid detergent fiber (ADF) and neutral detergent fiber (NDF) values of the samples were analyzed according to the procedures of Van Soest et al. [36].

### Sample collection and transcriptome sequencing

After stunning by electric shock, all goats were sacrificed by bleeding of carotid artery in a local abattoir at 6 h after receiving their morning feed on day 28. Ruminal contents were strained through a four-layer cheesecloth and immediately subjected to pH measurement. An aliquot (10 mL) of ruminal fluid was added to 1 mL of 5% HgCl_2_ solution and then stored at − 20 °C for the determination of SCFA concentrations. The whole liver was taken and washed in ice-cold phosphate-buffered saline (PBS; pH 7.4) until the PBS was clear. Pieces of approximately 1 × 4 cm in size, including periportal tissue and perivenous tissue, were cut from the liver and kept in tubes with Trizol buffer (Thermo Fisher Scientific, Nanjing, China). All liver samples were stored at − 80 °C until RNA extraction.

One piece per goat was used for the RNA extraction. Samples were homogenized in a Ball mill MM 400 (Retsch GmbH, Hahn, Germany). Total RNA was extracted by using Trizol buffer in accordance with the manufacturer’s instructions and was quantified by using a NanoDrop 1000 spectrophotometer (Thermo Fisher Scientific). RNA integrity was evaluated by using the RNA 6000 Assay Kit of the Agilent Bioanalyzer 2100 system (Agilent Technologies, Santa Clara, CA, USA). High-quality RNA (RNA Integrity number > 9.0) was processed by using an NEB Next Ultra RNA Library Prep Kit (New England Biolabs Inc., Ipswich, MA, USA) following the manufacturer’s instructions. Finally, 18 RNA-seq libraries were sequenced via paired-end chemistry (PE150) on an Illumina Hiseq X Ten platform (Illumina, San Diego, CA, USA) at Biomarker Technologies, Beijing, China.

### Rumen SCFA determination and analysis

The concentrations of ruminal SCFAs were determined by using a gas chromatograph (HP6890N, Agilent Technologies, Wilmington, DE, USA) according to the description of Yang et al. [37]. A two-tailed t-test was used in the analysis of rumen SCFA concentrations and rumen pH. Differences were considered significant when *p* < 0.05. These analyses were performed by using SPSS software package (SPSS Inc., Chicago, IL, USA).

### Transcriptome sequence analysis

Gene expression analysis: Quality of raw reads was checked by using FastQC. Low-quality reads were removed by using PRINSEQ v0.20.4 [38]*.* Reads shorter than 50 bps were discarded from the file. Goat genome assembly and gene expression analysis followed the protocol of Trapnell et al. [39]. In general, high-quality reads were mapped to the NCBI goat genome annotation release version 101 by using TopHat v2.1.0 [40]. Within the sample, the gene expression level was estimated and normalized to the FPKM by means of Cufflinks v2.2.1 [41]. Genes were identified to be expressed when FPKM > = 1 was found in at least one sample.

DEGs detection: Gene counts were obtained by means of HTSeq v0.6 [42] with mapped reads. The gene expression levels were compared across samples by means of the DeSeq2 package [43] with gene counts. Differences were considered significant when adjusted *p* < 0.05 and |log_2_(MNFC/LNFC)| > 1.

Systematic cluster analysis of samples: The hierarchical clustering method based on the euclidean distance metric and average linkage was applied in the systematic cluster analysis.

Pathway construction: the expressed genes were mapped to the following KEGG pathways by means of the pathview package in R [44]: (1) ureagenesis pathway (chx00220) and (2) the pathways related to energy metabolism, including FA biosynthesis (chx00061), FA degradation (chx00071), gluconeogenesis (chx00010), TCA cycle (chx00020), pyruvate metabolism (chx00620), propanoate metabolism (chx00640), butanoate metabolism (chx00650), glycerolipid metabolism (chx00561), and ketone synthesis (chx00072). Finally, these pathways were redrawn by using Photoshop CS 8.01 on the background of KEGG pathways [45].

Pathway difference analysis: the enrichKEGG function of the clusterProfiler package in R [46] was used to perform the KEGG pathway enrichment analysis. Differences were considered significant when *p* < 0.05.

Gene expression correlation analysis: SCC between pairs of genes was computed by means of R, in order to detect the correlations between the gene pairs. The correlation was considered as high when SCC > 0.8 and *p* < 0.05.

### RT-QPCR verification of target DEGs

An aliquot of 2000 μg RNA, random hexamer primers (Invitrogen, Shanghai, China) and moloney murine leukaemia virus (M-MLV) reverse transcriptase (Fermentas, Burlington, ON, Canada) were employed to synthesize the cDNA. RT-QPCR was performed by using the StepOne Plus real-time PCR system (Applied Biosystems, Den Ijssel, Netherlands) and SYBR-Green (Roche, Shanghai, China) for detection. Beta-actin (ACTB) was chosen as the stably expressed reference gene [47]. The primers of 40 DEGs listed in Table 2 were designed in this study by using Primer 5 and the available mRNA sequences in NCBI (Additional file 8). Amplification efficiencies of the primers were determined by means of a dilution series of epithelial cDNA. All samples were run in triplicate, and the data were analyzed according to the 2^−ΔΔCT^ method [48]. The identity and purity of the amplified products were checked by analysis of the melting curves obtained at the end of the amplification. Differences were considered to be significant when *P* < 0.05 was received in the two-tailed *t*-test. Finally, linear regression analysis was applied to identify the relationships between the RT-QPCR results and RNA-seq results.

## Supplementary information

**Additional file 1:** Number of clean reads, their quality scores and percentage of mapped reads to the goat genome for 18 RNA-seq samples.

**Additional file 2:** Differencially expressed genes identified in the RNA-seq analysis.

**Additional file 3:** The hierarchical cluster of gene expression profiles of 18 liver samples based on the euclidean distance metric and average linkage.

**Additional file 4:** Differentially expressed pathways detected by KEGG enrichment analysis.

**Additional file 5:** Additional file EC number, gene description, and expression levels of genes located on the pathways of hepatic energy metabolism and ureagenesis.

**Additional file 6:** Highly related genes along the pathways of hepatic energy metabolism and ureagenesis.

**Additional file 7:** RT-QPCR results of 40 selected genes.

**Additional file 8:** Primers used for the RT-QPCR.

## Data Availability

The transcriptome sequences are available on NCBI under BioProject PRJNA512146 with SRX5231298–5231315.

## Abbreviations

AA: Amino acid
ACTB: Beta-actin
ADF: Acid detergent fiber
ARG: Arginase
ASL: Argininosuccinate lyase
ASS1: Argininosuccinate synthase 1
ATP: Adenosine triphosphate
CL: Citrate lyase
CPS1: Carbamoyl phosphate synthase 1
CS: Citrate synthase
DEG: Differentially expressed gene
FA: Fatty acid
FASN: Fatty acid synthase
FBP: Fructose bisphosphatase
FPKM: fragments per kilobase of exon model per million reads mapped
G6PC3: Glucose-6-phosphatase
GDH: Glutamate dehydrogenase
GLS: Glutaminase
GLUL: Glutamate-ammonia ligase
GOT1: Glutamic-oxaloacetic transaminase 1
HAL: Histidine ammonia lyase
HMGCS1: 3-hydroxy-3-methylglutaryl-CoA synthase 1
JAK3: Janus kinase 3
KEGG: Kyoto encyclopedia of genes and genomes
LEP: Leptin receptor
LSU: Liver-synthesized urea
NADPH: Reduced nicotinamide adenine dinucleotide phosphate
NAG: N-acetyl-glutamate
NAGS: N-acetyl-glutamate synthase
NDF: Neutral detergent fiber
NFC: Non-fiber carbohydrate
OTC: Ornithine transcarbamylase
PC: Pyruvate carboxylase
PEP: Phosphoenolpyruvate
PPAR: Peroxisome proliferators activated receptor
PYCR1: Pyrroline-5-carboxylate reductase 1
RT-QPCR: reverse transcription quantitative PCR
SCC: Spearman correlation coefficient
SCFAs: Short-chain fatty acids
STAT3: Signal transducer and activator of transcription 3
TAG: Triacylglycerol
TCA: Tricarboxylic acid
α-KG: α-ketoglutarate
