# Supplementary figures and images for: Transcriptomic analyses suggest a dominant role of insulin in the coordinated control of energy metabolism and ureagenesis in goat liver
Source: BMC Genomics. 2019 Nov 14;20:854. doi: 10.1186/s12864-019-6233-9 (PMC6854773; doi:10.1186/s12864-019-6233-9)

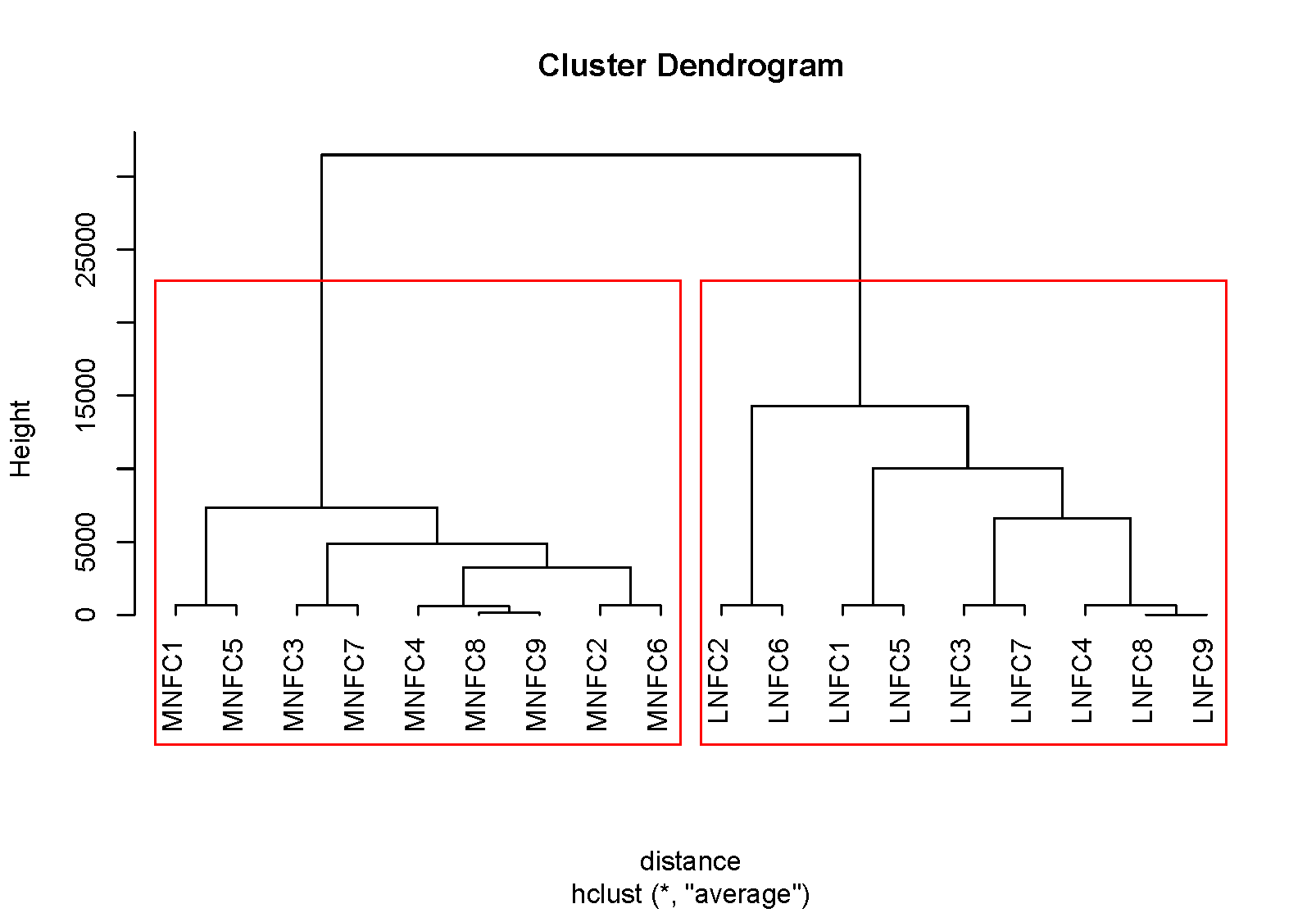

Supplement: Supplementary file 3 — Additional file 3: The hierarchical cluster of gene expression profiles of 18 liver samples based on the euclidean distance metric and average linkage. [file 12864_2019_6233_MOESM3_ESM.tiff]
